# IL‐6 signaling pathway contributes to exercise pressor reflex in rats with femoral artery occlusion in association with Kv4 activity in muscle afferent nerves

**DOI:** 10.14814/phy2.14935

**Published:** 2021-07-07

**Authors:** Qin Li, Lu Qin, Jianhua Li

**Affiliations:** ^1^ Heart and Vascular Institute The Pennsylvania State University College of Medicine Hershey PA USA

**Keywords:** blood pressure, dorsal root ganglion, exercise pressor reflex, IL‐6, Kv4 channels, muscle contraction, peripheral artery disease

## Abstract

Interleukin‐6 (IL‐6) via trans‐signaling pathway plays a role in modifying muscle sensory nerve‐exaggerated exercise pressor reflex in rats with ligated femoral arteries, but the underlying mechanisms are poorly understood. It is known that voltage‐gated potassium channel subfamily member Kv4 channels contribute to the excitabilities of sensory neurons and neuronal signaling transduction. Thus, in this study, we determined that 1) IL‐6 regulates the exaggerated exercise pressor reflex in rats with peripheral artery disease (PAD) induced by femoral artery ligation and 2) Kv4 channels in muscle dorsal root ganglion (DRG) neurons are engaged in the role played by IL‐6 trans‐signaling pathway. We found that the protein levels of IL‐6 and its receptor IL‐6R expression were increased in the DRGs of PAD rats with 3‐day of femoral artery occlusion. Inhibition of muscle afferents’ IL‐6 trans‐signaling pathway (gp130) by intra‐arterial administration of SC144, a gp130 inhibitor, into the hindlimb muscles of PAD rats alleviated blood pressure response to static muscle contraction. On the other hand, we found that 3‐day femoral occlusion decreased amplitude of Kv4 currents in rat muscle DRG neurons. The homo IL‐6/IL‐6Rα fusion protein (H. IL‐6/6Rα), but not IL‐6 alone significantly inhibited Kv4 currents in muscle DRG neurons; and the effect of H. IL‐6/6Rα was largely reverted by SC144. In conclusion, our data suggest that via trans‐signaling pathway upregulated IL‐6 in muscle afferent nerves by ischemic hindlimb muscles inhibits the activity of Kv4 channels and thus likely leads to adjustments of the exercise pressor reflex in PAD.

## Graphical abstract

Interleukin‐6 (IL‐6) regulates the exaggerated exercise pressor reflex in rats with peripheral artery disease (PAD) induced by femoral artery ligation. Kv4 channels in muscle dorsal root ganglion (DRG) neurons are engaged in the role played by IL‐6 trans‐signaling pathway.

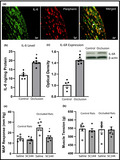

## 1 INTRODUCTION

With exercise, activation of the sympathetic nervous system increases arterial blood pressure (BP), heart rate (HR), myocardial contractility, and peripheral vasoconstriction (Sinoway et al., 1989; Victor et al., 1988). Two major mechanisms are thought to be involved in this process, namely central command (Goodwin et al., 1972; Waldrop et al., 1996) and exercise pressor reflex (McCloskey & Mitchell, 1972; Mitchell et al., 1983). The central command suggests that motor and sympathetic activation occur in parallel, that is, there is a volitional signal emanating from central motor areas leading to increased sympathetic nervous activity (SNA) during exercise. This system is linked to skeletal muscle metabolic needs via parallel brain activation of motor and autonomic centers. The exercise pressor reflex is mediated by group III and IV afferents arising from contracting skeletal in response to mechanical deformation and metabolic stimulation (Kaufman et al., 1984).

The exercise pressor reflex is exaggerated in peripheral artery disease (PAD) (Baccelli et al., 1999; Ritti‐Dias et al., 2011). In PAD patients, this reflex is a major determinant of why BP rises with exercise, that is, the BP rise during walking in the PAD patients is greater than that seen in healthy control subjects. Moreover, during static exercise the BP response with the affected limb is greater than that with the unaffected limb (Lorentsen, 1972). Notably, the augmented BP response during exercise is associated with higher incidence of cardiovascular diseases (Lewis et al., 2008) and less survival in asymptomatic normotensive subjects (Weiss et al., 2010) and in PAD patients (de Liefde et al., 2008). Nonetheless, the molecular mediators and underlying mechanisms alleviating the exercise‐exaggerated BP response in PAD need to be revealed.

PAD in human subjects is not solely a disease of large vessel obstruction, but it is a disease of large vessel obstruction in the setting of a chronic disease process (i.e., atherosclerosis) that is influenced by oxidative stress and inflammation (Signorelli & Katsiki, 2018). With atherosclerotic tissues the blood flow to the arteries of the lower extremities is decreased. During this process, proinflammatory cytokines (PICs) and related signaling molecular mediators are produced and released from numerous cells. Increased circulating and intramuscular levels of interleukin‐6 (IL‐6) are detected in PAD patients (Chaparala et al., 2009; Girn et al., 2007). The activity of exercise induces a greater increase in the levels of IL‐6 of the mixed venous blood in PAD patients than those levels in healthy age‐matched subjects (Palmer‐Kazen et al., 2009; Signorelli et al., 2003). Consistently, during the exercise ischemic insult also enhances the circulating IL‐6 levels compared with non‐ischemic exercise (Shill et al., 2017).

Three days of femoral artery occlusion increases products of oxidative stress in the hindlimb muscles of rats and activates inflammatory signaling pathways (Harms et al., 2017; Xing et al., 2018; Xing et al., 2018). IL‐6 also plays a role in regulating the exaggerated BP response to static exercise in PAD rats (Copp et al., 2015) likely via membrane‐bound IL‐6R or gp130 trans‐signaling pathways assembled by soluble form of IL‐6R (Jostock et al., 2001; Scheller et al., 2014; Wolf et al., 2014). Thus, we anticipated that the activity of IL‐6 signaling would be increased in muscle afferent nerves in involvement of the exercise pressor reflex in PAD rats.

Furthermore, A‐type voltage‐gated K^+^ (K_V_) channels are quintessential regulators of cellular excitability in the various tissues. IL‐6 can alter the expression and neuronal activity of voltage‐gated Kv subfamily Kv4 in regulating the excitability of dorsal root ganglion (DRG) neurons (Langeslag et al., 2014; Wang et al., 2007). Evidence also indicates that Kv4 channels, especially Kv4.3 mostly distributed in the small size of DRG neurons, play a critical role in the sensitization of primary afferent and nociceptive sensation (Cao et al., 2010; Chien et al., 2007; Conner et al., 2016; Kuo et al., 2017; Park et al., 2003; Viatchenko‐Karpinski et al., 2018). However, it is poorly known with respect to a linkage between IL‐6 and Kv4 channels in muscle DRG neurons, especially muscle afferent‐mediated exercise pressor reflex in PAD.

Accordingly, we hypothesized that the activity of IL‐6 trans‐signaling pathways in muscle afferent nerves is increased in rats with 3‐day of femoral artery occlusion and blocking IL‐6 trans‐signaling pathways attenuates the amplified BP response to static muscle contraction. We further hypothesized that the activity of Kv4 channels is a part of IL‐6 signaling pathways in regulation of muscle DRG neuronal excitation in PAD.

## 2 MATERIALS AND METHODS

### 2.1 Ethical approval

All experimental procedures were approved by the Institutional Animal Care and Use Committee of Penn State College of Medicine and were conducted in accordance with the National Institutes of Health Guidelines for the Care and Use of Laboratory Animals. Male Sprague–Dawley rats (4–6 weeks old) were housed in accredited temperature and ventilation controlled facilities with a 12:12‐hour light–dark cycle and ad libitum access to standard rat chow and water.

### 2.2 Femoral artery occlusion

**FIGURE 1:**
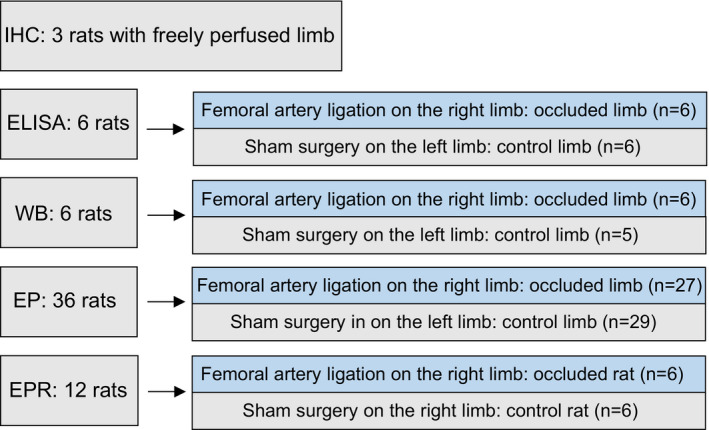
Showing the details of experimental groups and the number of rats used in this study. In vitro experiments, the femoral artery ligation was performed on the right limb and sham surgery was performed on the left limb in the same animals and the experimental groups were termed as occluded limb and control limb, respectively. In vivo experiments, the femoral artery ligation and sham surgery were performed on the right limb of individual rats and the experimental groups were termed as occluded rats and control rats. IHC: fluorescence immunohistochemistry; ELISA: enzyme‐linked immunosorbent assay; WB: western blot analysis; EP: electrophysiology; and EPR: exercise pressor reflex. The number of animals used in each experiment is shown in diagram

The rats were anesthetized by inhalation of an isoflurane–oxygen mixture (2%–5% isoflurane in 100% oxygen). The femoral artery on the right limb was surgically exposed, dissected, and ligated ~3 mm distal to the inguinal ligament as previously described (Li et al., 2020). As control, the left limb was dealt with the same procedure except the suture below the femoral artery not tied. This procedure was performed for *in vitro* experiments and the experimental groups were termed as occluded limb and control limb. For *in vivo* experiments, the femoral artery ligation and sham surgery were performed on the right limb of respective rats and the experimental groups were termed as occluded rats and control rats. Figure 1 containing a diagram shows more details of experimental groups and the number of rats used in this report. After the surgery, the rats were returned to the cage for regular housing for 3 days before experiments. Note that buprenorphine hydrochloride (0.05 mg/kg, subcutaneously) was administered prior to the surgery for postoperative pain relief. Following the surgery, the animals were kept in the surgery room for 2–3 h for observation, and then returned to the animal facility.

### 2.3 Fluorescence immunohistochemistry (IHC)

As described in our previous study (Xing et al., 2012), three rats with freely perfused limbs were anesthetized and transcardially perfused with 200 ml of ice‐cold saline (containing 1000 units heparin) followed by 500 ml of 4% ice‐cold paraformaldehyde in phosphate‐buffered saline (PBS). L4–L6 DRG tissues (DRGs) were dissected and immersed in the same fixative at 4°C for 2 h. The tissues were then stored in PBS containing 30% sucrose overnight. Then, 10 μm of DRG sections were obtained using a cryostat.

DRG sections were fixed in 4% of paraformaldehyde in PBS for 10 min at room temperature. After being washed with PBS, the tissues were permeabilized, blocked in 0.3% Triton X‐100 in PBS supplemented with 5% goat serum for 1 h, and then incubated with rabbit anti‐IL‐6 primary antibody (1:200, Novus #: NB600‐1131) overnight at 4°C. After washed in PBS, the sections were incubated with goat anti‐rabbit secondary antibody conjugated with fluorescein isothiocyanate (FITC) (1: 200, Thermo #31635) for 2 h at room temperature.

To examine the localization of IL‐6 within DRG neurons, the sections were incubated with a second primary antibody of mouse anti‐peripherin (1:200, Abcam #ab17999) to label the small diameter of neurons with thinly unmyelinated axons (C‐fibers) and then incubated with Alexa Fluor‐594 conjugated goat anti‐mouse IgG (1:1000, Thermo #A‐11‐32). After they were mounted, the sections were examined under a Nikon Eclipse 80i microscope and the images were stored digitally on a computer.

### 2.4 Enzyme‐linked immunosorbent assay (ELISA)

The protein levels of IL‐6 in the rat DRGs were assayed with ELISA. After euthanasia and decapitation, L4–L6 DRG tissues in both control limb (*n* = 6) and occluded limb (*n *= 6) were removed and dissected. The DRGs were lysed with ice‐cold radioimmunoprecipitation assay (RIPA) buffer (Sigma‐Aldrich #R0278) containing proteinase inhibitor cocktail (Sigma‐Aldrich #S8830). After this, the homogenate was centrifuged at 13,000 rpm for 25 min at 4°C to harvest the supernatant as the protein sample. After determination of the protein concentration with a bicinchoninic acid (BCA) assay kit (Thermo #23227), the samples were aliquoted and stored at −80°C for usage.

ELISA assay was performed at room temperature as instructed by manufacturer (Signosis). Briefly, the diluted samples and IL‐6 standard solution were distributed in the polystyrene 96‐well immunoplates (100 µl per well) and incubated for 2 h. After being thoroughly washed with wash buffer for three times, 100 µl of diluted biotin‐labeled antibody mixture was added to each well and incubated for 1 h. Then 100 µl of diluted streptavidin‐HRP conjugate per well was added and incubated for 45 min. Followed by washing, 100 µl of substrate per well was incubated for 45 min. The washing was repeated and 50 µl of stop solution per well was added. The optical density was examined using an ELISA reader (BioTek).

### 2.5 Western blotting analysis (WB)

As described previously (Qin et al., 2020), a total protein of rat L4 ‐ L6 DRG tissues in both control limbs (*n*=5) and occluded limbs (*n* = 6) were extracted. A quantity of 10 µg of protein was loaded in 10% Mini‐Protean TGX Precast gels (Bio‐Rad) after being boiled at 95°C for 5 min in SDS sample buffer, then electrophoretically transferred to polyvinylidene difluoride (PVDF) membrane. After blocking with 5% non‐fat milk in 0.1% Tween‐TBS buffer (TBST) for 1 h, the membrane was incubated with rabbit anti‐IL‐6R primary antibody at 4°C overnight and then incubated with a HRP‐conjugated anti‐rabbit secondary antibody (1:3000, Abcam #ab6721) at room temperature for 1 h. The immunoreactivity was visualized using an enhanced chemiluminescence system (Cell Signaling Technology #6883). The membrane was stripped and incubated with anti‐β‐actin primary antibody (1:3000, Abcam #ab8227) as the internal protein expression control. The optical densities of targeted bands were analyzed using the NIH Image J Software.

### 2.6 Examination of the exercise pressor reflex

The rats were anesthetized by inhalation and ventilated. The jugular vein and common carotid artery were cannulated, respectively, for fluids delivery and a pressure transducer connected to monitor of arterial BP. HR was calculated by beat to beat from the arterial pressure pulse. A catheter (PE10) was inserted into the femoral artery for SC144 (IL‐6 trans‐signaling pathway gp130 inhibitor) delivery. In the occluded rats, distal to the previously occluded site, the catheter was inserted into the femoral artery towards the distal end to deliver SC144 into the ischemic limb. During the experiments, baseline BP and fluid balance were maintained with a continuous infusion of saline and body temperature was also maintained at ~37°C with a heating pad.

Decerebration was performed to eliminate the effects of anesthesia on the reflex pressor response (Smith et al., 2001). Prior to the procedure, dexamethasone (0.2 mg, i.v.) was injected to minimize brain stem edema. A transverse section was made anterior to the superior colliculus and extending ventrally to the mammillary bodies and then all tissues from the rostral to the section were removed (Smith et al., 2001). Following this procedure, the anesthesia was withdrawn and a ventilator was applied to the rats, and 60 min were allowed before the experiment began.

A laminectomy procedure was performed to expose the lower lumbar and upper sacral portions of the spinal cord, and the peripheral ends of the transected L4 and L5 ventral roots were placed on platinum bipolar stimulating electrodes. Static muscle contractions were induced by electrical stimulation of the L4 and L5 ventral roots (30 s, 3× motor threshold with a duration of 0.1 ms at 40 Hz). The reflex BP and HR responses during muscle contraction were recorded in control rats and occluded rats (*n* = 6 in each group). SC144 was resolved in saline and 125 ng/kg in 0.1–0.15 ml of injection volume was given intra‐arterially according to the rat's body weight. The duration of the injection was 1 min, and 20 min were allowed between injections. Injection of the same volume of saline was used as vehicle control.

### 2.7 Electrophysiology

#### 2.7.1 Labeling of hindlimb muscle afferent DRG neurons

Briefly as described (Li et al., 2020; Xing et al., 2012), 2 days before femoral artery ligation was performed an incision in the calf area of hindlimbs was made and the gastrocnemius muscle was exposed in thirty‐six rats following they were anesthetized. The retrograde tracer lipophilic dye 1, 1’‐dioctadecyl‐3, 3, 3’, 3’‐tetramethylindocarbocyanine perchlorate (DiI, 60 mg/ml) was injected into the white portion of the gastrocnemius muscle. A total volume of 1 µl DiI was administered at different locations, with the needle left in the muscle for 1 min to prevent the tracer leakage. Then, the rats were returned to their cages to wait for the DiI transported to the DRGs and label muscle DRG neurons.

#### 2.7.2 Culture of DRG neurons

As described (Li et al., 2020), the rats were euthanatized and decapitated, L4–L6 DRGs of control limbs and occluded limbs were removed and dissected, immediately transferred into ice‐cold Hank's balanced salt solution. After freeing from the connective tissues, the ganglia were enzymatically digested and dissociated in Earle's balanced salt solution (Sigma‐Aldrich) containing collagenase Type D (0.6 mg/ml; Roche), trypsin (0.30 mg/ml; Worthington), and DNase (0.1 mg/ml; Alfa Aesar), followed by shaking for 40 min at 34°C. The dissociated neurons were seeded on 10% poly‐L‐lysine–coated coverslips (Dia^#^ 8mm) in a 35‐mm culture dish containing 2 ml of DMEM medium (Thermo) supplemented with 10% FBS, 1% glutamine, and 1% penicillin–streptomycin. Then the neurons were cultured at 37°C with 5% CO2, 95% air in a cell culture incubator (VWR). Then, IL‐6, homo IL‐6/IL‐6Rα fusion protein (H. IL‐6/6Rα) and SC144 were added to the culture solution in this experiment, respectively, as detailed in the results. The dosages of those agents were selected according to our pilot experiments and previous reports (Fang et al., 2015; Liu, Chen, et al., 2019; Xia et al., 2015). Then, the patch recording was completed within 6 to 48 h after DRG neurons were dissociated.

#### 2.7.3 Recording of K^+^ currents and action potential (AP) firing

Muscle DRG neurons (Dil‐positive neurons) were first identified under an inverted microscope with a fluorescent filter (Nikon TE2000), and images were displayed on a video monitor. K^+^ currents and AP firing of rat muscle DRG neurons (cell diameters ≤35 µm) were recorded in the whole‐cell configuration using a MultiClamp 700B amplifier supplied with Digitizer 1440A (Axon Inc). Signals were acquired with pClamp10.1 and analyzed with pClampfit10.7 software.

The extracellular solution contained (in mM): 110 choline chloride, 5 KOH, 1 MgCl2, 20 tetraethylammonium (TEA), 10 HEPES, 2 CdCl2, and 10 D‐glucose (pH 7.4, osmolality 310 mosm). The electrode was filled with a solution containing (in mM): 120 KCl, 2.5 MgCl_2_, 10 EGTA, 10 HEPES, 0.3 Li‐GTP, 2 MgATP, and 1 CaCl_2_ (pH 7.3 adjusted with KOH, osmolality 290 mosm).

Holding at −65 mV, after the seals (2~8 GΩ) were obtained with 2~4 MΩ resistance of glass electrodes filled with internal solution, the whole‐cell configuration was applied. In voltage‐clamp mode, two separate protocols were used to record TEA resistant A‐type‐K^+^ channel currents (TEA‐R‐IKA) in muscle DRG neurons as previously reported (Phuket & Covarrubias, 2009; Qian et al., 2009). Total K^+^ currents (IKA_total_) were recorded with the *protocol a*: a 1‐sec conditioning pulse of −100 mV prior to 500 ms step depolarizations from −100 mV to 60 mV with 10 mV increments per step. The *protocol b was* similar to *protocol a* except of 1‐sec conditioning pulse of −30 mV for the delayed rectifier K^+^ currents (IKA_DR_). The recording currents were filtered at 2 kHz and sampled at 10 kHz. Voltage errors were minimized by 80% series resistance compensation and linear leak subtraction was used for all recordings. Then TEA‐R‐IKA (=IKA_total_ − IKA_DR_) was obtained. A quantity of 20 μM of TEA was applied to the external solution to block the activity of TEA sensitive K^+^ currents.

AP of muscle DRG neurons was determined by whole cell current‐clamp recording. The extracellular solution contained (in mM): 150 NaCl, 5 KCl, 2 CaCl_2_, 2 MgCl_2_, 10 HEPES, and 10 D‐glucose (pH 7.4 adjusted with NaOH, osmolality 330 mosm). The electrodes were filled with a solution containing (in mM): 135 K^+^‐ gluconate, 5 KCl, 2 MgCl_2_, 2 CaCl_2_, 5 EGTA, 10 HEPES, 0.3 Na‐GTP, 2 MgATP (pH 7.3 adjusted with KOH, osmolality 310 mosm). In voltage‐clamp mode, the whole‐cell configuration was applied, then switched to current‐clamp mode, and AP generation was stimulated with a 1‐sec step currents protocol: −50pA to 500pA with an increments of 10pA, and then the AP firing threshold and frequency were analyzed.

All the chemicals stored in the stock solutions were diluted in extracellular solution immediately before being used and individually held in a series of independent syringes of the pressurized VC3‐8MP perfusion system (ALA Inc.) with outlet tip. The distance from the outlet tip mouth to the neuron examined was within 100 µm. The DRG neurons in the recording chamber were continuously bathed in extracellular solution.

### 2.8 Statistical analysis

Unless specified, the experimental data included in this study were presented as mean ±standard error. A paired *t*‐test was used to compare the expression of IL‐6 and IL‐6R in DRG tissues of control and occluded limbs as well as Density_60mV_ when AmmTX3 was applied. One‐way ANOVA was applied to compare the difference in responses of MAP and muscle tension, and Density_60mV_ in other groups. As appropriate, post‐hoc analysis with Tukey's tests was applied to compare the difference between specific groups. All statistical analyses were performed using SPSS v26, and the significant differences were considered at *p* < 0.05.

## 3 RESULTS

### 3.1 Levels of IL‐6 and IL‐6R expression were increased in DRGs of occluded limbs

**FIGURE 2:**
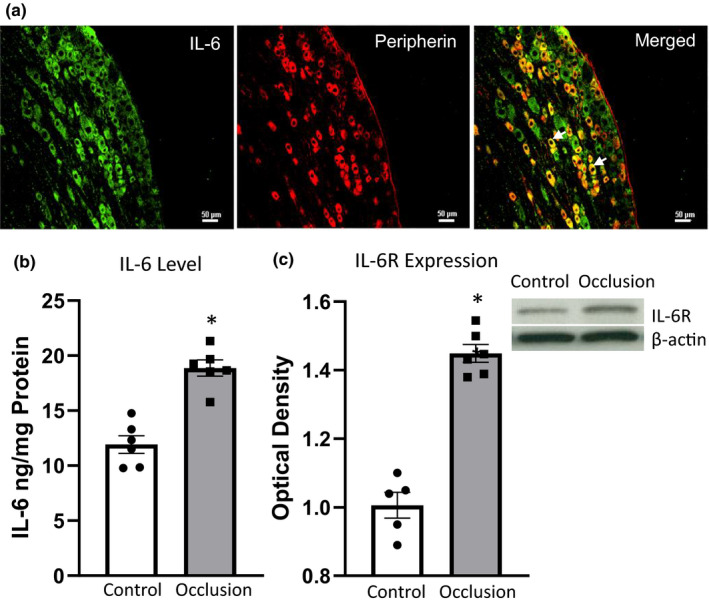
The effect of 3‐day femoral artery occlusion on IL‐6 and IL‐6R in rat DRGs. (a) The dual IHC technique was employed to determine if there was the distribution of IL‐6 within DRG neurons dissected from freely perfused rats (*n* = 3). IL‐6 and peripherin staining were, respectively, characterized by fluorescent green and red color. Arrows illustrate the merged images within the same DRG neurons, indicating that IL‐6 and peripherin appear within the same DRG neurons. Scale bar =50 µm. (b) A histogram showing that IL‐6 protein level assayed using ELISA in DRGs of occluded limbs was elevated as compared with control limbs (*n* = 6 in each group; *p < 0.05 vs. control). (c) The averaged data of IL‐6R relative expression in rat DRGs assessed by western blot analysis, and representative bands of IL‐6R and β‐actin as the internal protein control. Protein expression of IL‐6R was upregulated in DRGs of occluded limbs (*n* = 6). *p < 0.05 vs. control limbs (*n* = 5). Dots/spots indicate individual data and it is the same in each figure

The dual IHC technique was first employed to determine if there was the distribution characteristics of IL‐6 within DRG neurons from freely perfused rats (*n* = 3). As shown in Figure 2a, IL‐6 was mostly present in DRG neurons with diameter size of ~15–45 μm, and it was observed to co‐localize with peripherin positive cells in DRGs. Using ELISA (Figure 2b), we further observed that 3‐day of femoral artery occlusion significantly increased the IL‐6 protein levels in the DRGs of occluded limbs as compared with the controls (*p* < 0.05, *n* = 6 in each group). In addition, the levels of IL‐6R, assayed with WB (Figure 1c), were examined in DRGs, and the optical density of the targeted band signal in DRGs of occluded limbs (*n* = 6) was ~1.4‐fold greater than that in control limbs (*n* = 5, *p* < 0.05, occlusion *vs*. control).

### 3.2 Blocking IL‐6 signaling pathways using SC144 attenuated the exaggerated BP response to static muscle contraction in occluded rats

**FIGURE 3:**
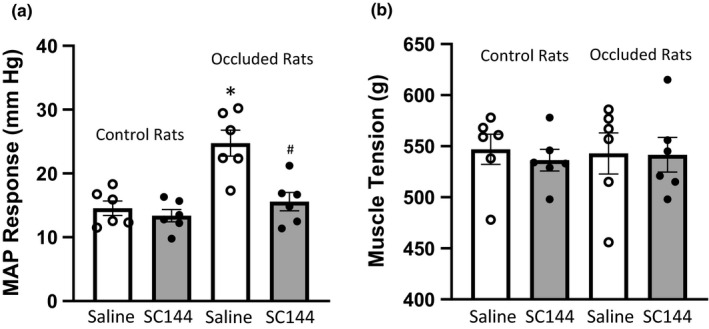
The effect of SC144 on the pressor response in rats. Static muscle contraction‐mediated pressor response was evoked by electrical stimulation of the L4 and L5 ventral roots. (a) Arterial injection of SC144 (gp130 inhibitor, 125 ng/kg) attenuated BP response induced by muscle contraction in occluded rats (*n* = 6), but not in control rats (*n* = 6). *p < 0.05 *vs*. control rats as saline was given; and #*p* < 0.05, SC144 *vs*. saline in occluded rats. (b) No significant difference in muscle development tension was observed before and after injection of SC144 in control rats and occluded rats

Furthermore, we examined the effect of SC144 (gp‐130 inhibitor) on the pressor response to static muscle contraction in both control rats and occluded rats. Baseline MAP was 95 ± 8 mmHg in control rats; 98 ± 9 mmHg in occluded rats with 3‐day of femoral artery occlusion (*p* > 0.05 between two groups). As shown in Figure 3a, intra‐arterial injection of 125 ng/kg SC144 was observed to significantly attenuate the BP response to static muscle contraction in the occluded rats, but not in the control rats. In control rats, peak MAP response was 15 ± 2 mmHg after saline and 13 ± 1 mmHg after 125 ng/kg SC144 (*n* = 6; *p* > 0.05 vs. saline). In occluded rats, peak MAP response was 25 ± 3 mmHg after saline and 15 ± 2 mmHg after 125 ng/kg SC144 (*n* = 6; *p* < 0.05 vs. saline). As well, as shown in Figure 3b there was no significant difference in developed muscle tension after using saline and SC144 in control rats and occluded rats. In addition, there were no significant differences in baseline HR between control rats and occluded rats (386 ± 28 bpm in control rats; 395 ± 35 bpm in occluded rats; *p* > 0.05 between two groups). No significant difference was observed in HR response to muscle contraction after SC144 in either control rats or occluded rats. That is, HR response was 19 ± 3 bpm after saline and 18 ± 3 bpm after SC144 in control rats (*p* > 0.05 between two groups); and 21 ± 3 bpm after saline and 17 ± 5 after SC144 in occluded rats (*p* > 0.05 between two groups).

### 3.3 Activity of Kv4 channels and AP in rat muscle DRG neurons

**FIGURE 4:**
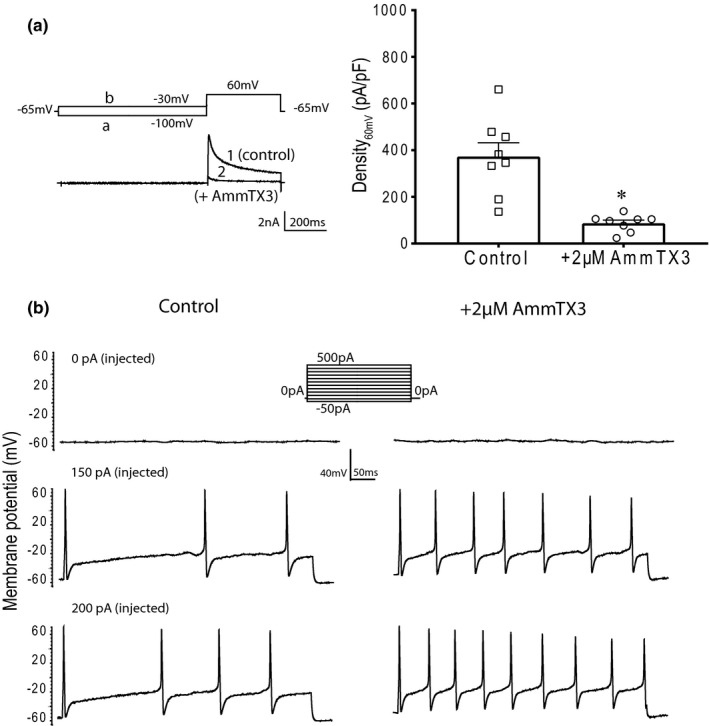
Engagement of Kv4 channels in neuronal activities of rat DRGs innervating the hindlimb muscles. (a): A‐type‐K^+^ channel currents (TEA‐R‐IKA) in Dil‐positive DRG neurons. The left panel shows the representative traces of TEA‐R‐IKA in rat muscle DRG neurons. *Protocol a* was used to record the total K^+^ currents (IKA_total_), and *Protocol b* for the delayed rectifying current (IKA_DR_), TEA‐R‐IKA = IKA_total_ − IKA_DR_ calculated using pClampfit 10.7. Trace 1 and trace 2 were before and after application of 2 μM of AmmTX3 for 5 min. The right panel shows the Density_60mV_ of TEA‐R‐IKA in muscle DRG neurons was decreased when treated with 2 μM AmmTX3. *p < 0.05, comparing to the untreated neurons; the number of recorded neurons = 8. (b): The representatives of action potential (AP) firing traces in rat muscle DRG neurons when step‐current protocol applied, −50 pA to 500 pA in 10 pA increments per step. The protocol was shown as 50 pA increments per step. The frequency of AP evoked by the injected current was increased in DRG neurons after application of AmmTX3

TEA‐R‐IKA in Dil‐positive DRG neurons (cell dimeter ≤35mm) was examined. According to the previous reports (Maffie et al., 2013; Vacher et al., 2002), 2 µM of AmmTX3 toxin (a specific blocker to Kv4 channels) was used to block the activity of Kv4 channels in rat muscle DRG neurons. As shown in Figure 4a, with treatment of 2 µM of AmmTX3 for 5 min, the current density of TEA‐R‐IKA when depolarizing to 60mV from −100mV (Density_60mV_), was reduced by 76 ± 2% from 373 ± 59 pA/pF to 87 ± 13 pA/pF (*p* < 0.05 between two groups, *n*= 8, the number of DRG neurons). This indicates that TEA‐R‐IKA mostly represents the activity of Kv4 channels. On the other hand, the AP firing frequency in muscle DRG neurons evoked by injected currents was increased after 2 µM of AmmTX3 application (Figure 4b). That is, frequency of AP was increased from 6.17 ± 1.87 /sec of control to 11.83 ± 1.80 /sec at 200pA of injected current (*p* < 0.05 between control and AmmTX3, *n* = 6).

### 3.4 IL‐6 inhibited Kv4 currents in rat muscle DRG neurons via trans‐signaling pathways

**FIGURE 5:**
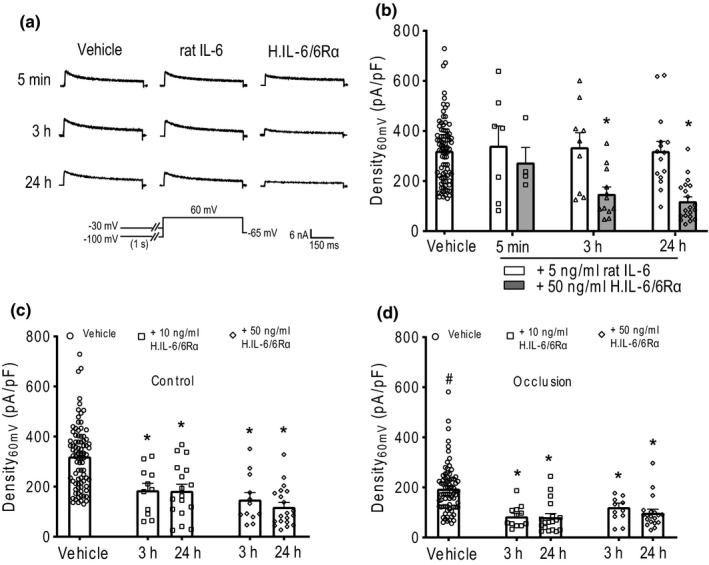
The effect of IL‐6 on Kv4 currents in rat muscle DRG neurons. (a) The representative traces of TEA‐R‐IKA in muscle DRG neurons of the control limbs. (b) Histogram of Density_60mV_ of TEA‐R‐IKA in muscle DRG neurons of the control limbs when treated with 5 ng/ml IL‐6 and 50 ng/ml homo IL‐6/IL‐6Rα fusion protein (H. IL‐6/6Rα) at different time courses. *p < 0.05, comparing to the vehicle‐treated‐neurons. (c and d) Histograms of Density_60mV_ of TEA‐R‐IKA in muscle DRG neurons of the control and the occluded limbs when H. IL‐6/6Rα was applied for 3 h and 24 h. *p < 0.05, respectively, comparing to the vehicle‐treated neurons for each group of control limbs and occluded limbs. #*p* <0.05 between control group and occlusion group. Dots/spots indicate individual data and a total number of DRG neurons in each group

Then, we determined the effect of IL‐6 on Kv4 currents in rat Dil‐positive DRG neurons. According to the prior reports (Fang et al., 2015; Liu, Chen, et al., 2019; Xia et al., 2015), we first examined the effect of IL‐6 alone and homo IL‐6/IL‐6Rα fusion protein (H. IL‐6/6Rα, stimulating IL‐6 trans‐signal complex) on Kv4 currents over time in muscle DRG neurons of control limbs. As shown in Figure 5a,b, IL‐6 applied for 5 min, 3 h and 24 h had no effect on Density_60mV_ of TEA‐R‐IKA in rat muscle DRG neurons. However, H. IL‐6/ 6Rα applied for 3 h and 24 h significantly attenuated Density_60mV_ of TEA‐R‐IKA in rat muscle DRG neurons; and Density_60mV_ was, respectively, decreased to 148 ± 28 pA/pF (*n* = 12) and 119±18 pA/pF (*n* = 20) (*p* <* 0*.05, compared to the vehicle‐treated muscle neurons: 332 ± 18 pA/pF, *n* = 85) (Figure 5b). In addition, the inhibitory effect on Kv4 currents was observed with application of a lower dose of H. IL‐6/6Rα to muscle DRG neurons incubated for 3 h or 24 h (Figure 5c). The results indicated that IL‐6 via trans‐signaling pathway of IL‐6 played an inhibitory role in regulating activity of Kv4 currents in muscle DRG neurons.

Furthermore, we determined the effect of H. IL‐6/6Rα on the muscle DRG neurons of occluded limbs (Figure 5d). As shown in Figure 5c,d, without H. IL‐6/6Rα Density_60mV_ of TEA‐R‐IKA in muscle neurons of occluded limbs became smaller than that in control limbs: 195±11 pA/pF (*n*=75) after occlusion and 341±79 pA/pF (*n* = 85) in controls (*p* < 0.05 between the two groups). Nonetheless, 50 ng/ml H. IL‐6/6Rα significantly attenuated Density_60mV_ of TEA‐R‐IKA in muscle neurons of occluded limbs to 110 ± 15 pA/pF (*n* = 11) and 98 ± 14 pA/pF (*n* = 12) after incubating for 3 h and 24 h (*p* < 0.05, compared to the vehicle‐treated muscle neurons of occluded limbs).

**FIGURE 6:**
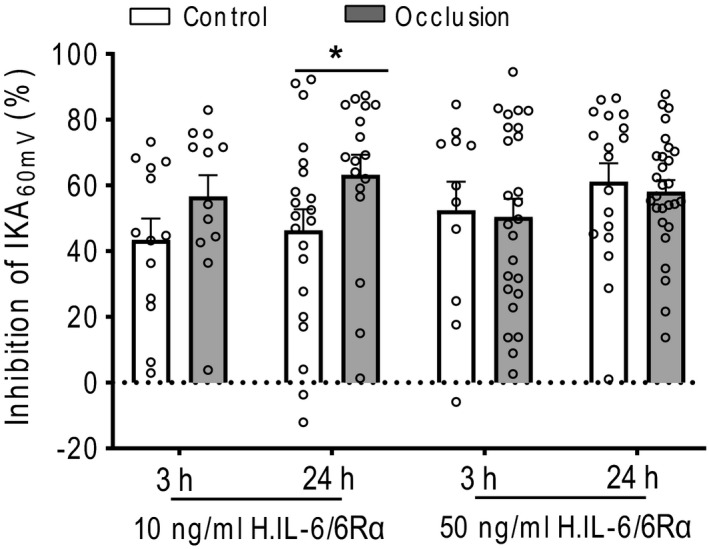
The inhibitory efficiency of H. IL‐6/ 6Rα on Density_60mV_ of TEA‐R‐IKA in muscle DRG neurons of control limbs and occluded limbs. The mean Density_60mV_ of the vehicle‐treated muscle neurons as the baseline to calculate the inhibitory efficiency in each group. *p < 0.05, occlusion (*n* = 17) vs. control (*n* = 21) at the same experimental conditions. The number of recorded neurons is indicated by dots in each group

Likewise, 10 ng/ml H. IL‐6/6Rα also significantly decreased Density_60mV_ of TEA‐R‐IKA in muscle neurons of occluded limbs. In addition, it was noticed that the % inhibitory effect of H. IL‐6/6Rα on Density_60mV_ of TEA‐R‐IKA (Figure 6), that is, 10 ng/ml over 24 h of application, appeared to be greater in muscle DRG neurons of occluded limbs (64 ± 6%, *n* = 17) than that in control limbs (46 ± 6%, *n* = 21; *p *< 0.05 between the two groups). These results indicated that femoral artery occlusion led to activity of Kv4 channels in muscle DRG neurons to a less degree, and/or that IL‐6 was likely to have a greater inhibition on Kv4 currents in rat muscle DRG neurons following femoral artery occlusion through trans‐signaling pathways of IL‐6. No significant difference in TEA‐R‐IKA was found after 50 ng/ml H. IL‐6/ 6Rα at time point of 3 h and 24 h in each of control group and occlusion group.

### 3.5 SC144 reverted the inhibition of H. IL‐6/6Rα on Kv4 currents in rat muscle DRG neurons

**FIGURE 7:**
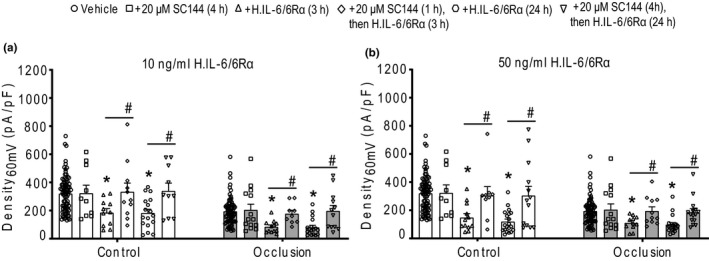
The effect of SC144 on Kv4 currents in rat muscle DRG neurons. (a and b) Histograms of Density_60mV_ of TEA‐R‐IKA in muscle DRG neurons of both control limbs and occluded limbs when treated with 20 μM SC144 together with 10 ng/ml H. IL‐6/6Rα or 50 ng/ml H. IL‐6/6Rα. *p < 0.05, comparing to the vehicle‐treated DRG neurons, respectively, in the control and occlusion groups. #*p* < 0.05 between DRG neurons treated with H. IL‐6/6Rα alone and DRG neurons treated with SC144 plus H. IL‐6/6Rα for each of control group and occluded group. Dots/spots indicate individual data and a total number of DRG neurons in each group

To further clarify involvement of IL‐6 via trans‐signaling pathway, 20 µM of SC144 was used onto muscle DRG neurons prior to H. IL‐6/6Rα. As shown in Figure 7a,b, SC144 did not affect amplitude of Kv4 currents in muscle DRG neurons of the control and occluded limbs after its application per se. However, incubating with 20 µM SC144 for 1 h largely reverted the inhibition of IL‐6/6Rα on Kv4 currents in muscle DRG neurons of the control and occluded limbs (Density_60mV_: 333 ± 62 pA/pF in controls, *n* = 9; and 178 ± 24 pA/pF after occlusion, *n* = 9; *p* < 0.05 when, respectively, comparing to those in the muscle neurons treated with 10 ng/ml H. IL‐6/6Rα for 3 h in each group). Likewise, Figure 7a shows that 20 µM of SC144 also alleviated the inhibition of 10 ng/ml H. IL‐6/6Rα on Kv4 currents in muscle DRG neurons of the control and occluded limbs following application of H. IL‐6/6Rα for 24 h. The similar effects of SC144 were also observed in muscle DRG neurons treated with 50 ng/ml of H. IL‐6/6Rα in both the control and occluded limbs (Figure 7b).

## 4 DISCUSSION

In the present study, using a PAD rat model induced by 3‐day of femoral artery ligation, we demonstrated that 1) femoral artery occlusion increases IL‐6 and its receptor IL‐6R expression in rat DRGs; 2) the smaller amplitude of Kv4 currents appears in muscle DRG neurons of occluded limbs; 3) IL‐6 inhibits the activity of Kv4 currents in muscle DRG neurons via IL‐6 trans‐signaling pathways; and 4) SC144, inhibiting gp130 responsible for IL‐6 trans‐signaling pathways, largely reverts the inhibitory effect of IL‐6 on Kv4 currents in rat muscle DRG neurons, and alleviates the exaggerated BP response to static muscle contraction in occluded rats.

In general, in various tissues IL‐6 generates the IL‐6/IL‐6R/gp130 complex and activates the downstream signaling cascades to play a role by its binding with membrane IL‐6R receptor (mIL‐6R) (classic signaling), or binding with the extracellular soluble IL‐6R (sIL‐6R) (trans‐signaling) assembled with gp130 receptor on the cell membrane (Rothaug et al., 2016; Simpson et al., 1997).

In a previous study, the role played by IL‐6 with sIL‐6R and its inhibitor in modulating BP response to muscle contraction was examined (Copp et al., 2015). Results of this previous report showed that arterial injection of IL‐6 and sIL‐6R significantly increased the BP response to static contraction as compared with controls. This previous study also showed that arterial injection of sgp130, which inhibits the effect of the IL6‐sIL‐6R complex, significantly alleviated the exaggerated exercise pressor reflex in rats with an occluded femoral artery. These findings suggest that IL6‐sIL‐6R has an endogenous role in modifying the exercise pressor reflex in PAD rats. Nonetheless, it is interesting to determine the precise mechanisms at cellular levels for how IL‐6 plays a role in regulating the exercise pressor reflex in PAD rats.

In the present study, we first identified that within DRG neurons with a diameter of 15–45 μm IL‐6 co‐localized with peripherin (Figure 2a), a marker to label the small diameter of neurons supplying thinly unmyelinated C‐fibers (Saveri et al., 2020; Sleigh et al., 2017). In addition, we found that the protein levels of both IL‐6 and IL‐6R were increased in the DRGs of PAD rats after femoral artery was occluded for 3 days (Figure 2b,c). The result supports the notion that upregulated IL‐6 is involved in thin fiber of muscle afferent nerve‐mediated the exaggerated exercise pressor reflex in PAD rats. Similarly, the expression levels of IL‐6, IL‐6R, and gp130 were elevated in the superficial spinal cord and DRGs of various pain models when compared to the sham controls (Zhou et al., 2016).

SC144, a quinoxalinhydrazide derivative, binds gp130 receptor and induces gp130 phosphorylation, deglycosylation, and conformational changes, then destructs the IL‐6/IL‐6R/gp130 complex and the transduction signaling cascade (Xu et al., 2013). SC144 has been widely used to inhibit the IL‐6 signaling pathways in growth tissues and the sensory nervous system‐mediated cancer pain (Heo et al., 2016; Zhang et al., 2019). Recent studies further suggest that SC144 inhibits IL‐6 signal and effectively blocks subsequent intracellular p38‐MAPK, JNK pathways in the rat DRG neurons, thereby alleviates mechanical hyperalgesia and cold hypersensitivity seen in cancer pain (Liu, Sun, et al., 2019; Zhao et al., 2019). Therefore, in the present study, we used SC144 to inhibit gp130 and examined its effect on the pressor response to static muscle contraction. We found that administration of SC144 into the arterial blood supply to the occluded limb attenuated the exaggerated BP response to muscle contraction in PAD rats, but had minimal effects on the BP response in control rats (Figure 3). This is in line with results of the previous report showing that intra‐arterial injection of sgp130 (inhibitor to IL‐6/sIL‐6) into the hindlimb can attenuate the augmented exercise pressor reflex in PAD rats (Copp et al., 2015).

K^+^ channels are quintessential regulators of the electrical excitability in the central and peripheral nervous system. They participate in the process of the resting membrane potential and the AP such as the shape of AP and repolarization, thereby playing a critical role in sensory nerve‐mediated functions, that is, mechanical and thermal sensation, as well as nociception under diseased conditions (Tsantoulas & McMahon, 2014). Numerous reports have demonstrated that a wide range of K^+^ channels in DRG neurons contribute to the excitabilities of nociceptors and nociceptive sensation (Smith, 2020). Among these K^+^ channels, evidence shows that the expression of Kv4 channels in DRG neurons are downregulated along with a hypersensitive nociceptive response either in the inflammation pain models or in nerve injury pain models (Cao et al., 2010; Chien et al., 2007; Conner et al., 2016; Kuo et al., 2017; Viatchenko‐Karpinski et al., 2018). Additionally, Kv4 channels contain Kv4.1, Kv4.2, and Kv4.3 α subunits; and Kv4.1, Kv4.3 subunits are predominant in DRG neurons, especially Kv4.3 in small size of DRG neurons, whereas Kv4.2 subunit is mainly distributed in the central nervous system (Cheng et al., 2016; Chien et al., 2007; Huang et al., 2005; Phuket & Covarrubias, 2009).

Because the exercise pressor reflex is mediated by thin‐myelinated group III/Aδ fibers and unmyelinated group IV/C fibers arising from contracting skeletal muscle (Kaufman et al., 1984), which mostly correspond to the small to medium size of DRG neurons (Basbaum & Woolf, 1999; Lawson & Waddell, 1991). Thereafter, we labeled rat muscle DRG neurons with fluorescent tracer Dil and examined the activities of Kv4 channels in Dil‐positive DRG neurons. The subthreshold A‐type‐K^+^ currents TEA‐R‐IKA was observed in the muscle DRG neurons with diameter ≤35 µm (Figure 4a). This result is consistent with the previous findings in rat DRG neurons (Chien et al., 2007; Phuket & Covarrubias, 2009). We further found that blocking Kv4 channels using AmmTX3 increased the AP frequency elicited by injected currents in muscle DRG neurons (Figure 4b). Generally, when AP is generated the more firing frequency and/or has the lower current threshold to a stimulus it indicates that neuronal cells are more excitable or more sensitive the stimulus (Barnett & Larkman, 2007). Thus, our results demonstrated that a blockade of Kv4 channels enhanced the excitabilities of muscle DRG neurons. The data further suggested that the activity of Kv4 channels (as Kv4 currents in this study) mainly contributed to a component of TEA‐R‐IKA in muscle DGR neurons and participated in the neuronal excitabilities. Therefore, our current results suggest the role played by Kv4 channels in muscle DRG neurons in regulating neuronal excitabilities.

IL‐6 has a sensitizing effect on afferent nerves and stimulates neurotransmitter release from nerve terminals, also it can sensitize activities of DRG neurons in regulating pain (Hoheisel et al., 2005; Yan et al., 2012). Chronic exposure to IL‐6 increases the excitabilities of rat DRG neurons linked to K^+^ currents (Wang et al., 2007). Thus, it was anticipated that Kv4 channels in muscle DRG neurons are potentially involved in signaling pathways of IL‐6 in regulation of over‐sensitized muscle afferent nerves by femoral artery occlusion. Accordingly, in the present study, we examined if IL‐6 played an inhibitory role in the activities of Kv4 channels in rat muscle DRG neurons. We found that IL‐6 alone did not affect Kv4 currents in muscle DRG neurons with even its application for 24 h (Figure 5). In contrast, H. IL‐6/6Rα fusion protein significantly reduced amplitude of Kv4 currents in muscle DRG neurons after incubating over 3 h (Figure 5). Notably, 10 ng/ml of H. IL‐6/6Rα decreased Kv4 currents in muscle DRG neurons of occluded limbs to a greater degree and the different effect was not observed with 50 ng/ml of H. IL‐6/6Rα (Figure 6). This result indicates that a higher dose of H. IL‐6/6Rα was likely to reach a peak stimulation on IL‐6R receptors in muscle DRG neurons of both control and occlusion groups. Nonetheless, via trans‐signaling pathway IL‐6 inhibits the activity of Kv4 in rat muscle DRG neurons and this is likely a part of mechanisms leading to the exaggerated exercise pressor reflex in PAD rats.

Moreover, with application of a gp130 inhibitor SC144 onto muscle DRG neurons, we observed that SC144 largely reversed the inhibition of H. IL‐6/6Rα on the Kv4 currents in muscle DRG neurons of both control limbs and occluded limbs (Figure 7). This result further clarified that IL‐6 inhibits the activities of Kv4 channels in rat muscle DRG neurons through IL‐6/sIL‐6R/gp130 trans‐signaling pathways, which provided additional evidence showing that IL‐6 plays an inhibitory role in regulating the activity of Kv4 channels in rat muscle DRG neurons through its trans‐signaling pathways. Remarkably, in the whole animal preparation of the present study, intra‐arterial injection of SC144 into the occluded hindlimb was observed to attenuate the muscle afferent nerve‐activated exaggerated exercise pressor reflex in PAD rats. Overall, it is well reasoned for the role played by IL‐6 in regulating this autonomic reflex likely via Kv4 in muscle afferent neurons of PAD rats.

Finally, it should be noted that there are differences in the results of in vitro and in vivo experiments. Intra‐arterial injection of SC144 significantly attenuated the exaggerated BP response in occluded rats, but slightly changed the BP response in control rats. However, application of SC144 onto DRG neurons decreased the effects of H. IL‐6/6Rα on Kv4 currents in muscle DRG neurons of both the control and occluded groups. The differences are likely due to that endogenous activation of IL‐6 signal pathways was lacking in control rats in our current in vivo experiment. In contrast, in vitro experiment application of H. IL‐6/6Rα directly activated the IL‐6 signal pathways. This seems to be explained by the previous study demonstrating that sgp130 can reverse the increase of BP response induced by exogenous IL‐6/sIL‐6 application in control rats (Copp et al., 2015). Nonetheless, the levels of IL‐6 and IL‐6R were increased by 3‐day of femoral artery occlusion and this exaggerated endogenous IL‐6 signal pathways. Thus, we observed that the effects of SC144 on reflex BP response in occluded rats and the effects of H. IL‐6/6Rα on Kv4 currents in muscle DRG neurons of occluded group.

In conclusion, we found increases in the levels of IL‐6 and expression of its receptor IL‐6R in DRGs of PAD rat, which are likely to decrease the activities of Kv4 channels in muscle DRG neurons via IL‐6 trans‐signaling pathways and make IL‐6‐Kv4 channel signaling a specific molecular mechanism leading to the exaggerated pressor reflex in PAD.

## CONFLICT OF INTEREST

The authors declare no conflict of interest.

## AUTHOR CONTRIBUTIONS

Q Li contributed to data collection and analysis of electrophysiology data, and drafted the manuscript. L Qin participated in analysis of western blot and blood pressure data and drafting the manuscript. J Li designed experiments, oversaw performance of the experiments, and data analysis and revised the manuscript. All authors approved the final version of the manuscript submitted for publication.
